# Immunogenetic Epidemiology of Motor Neuron Diseases in 14 Continental Western European Countries

**DOI:** 10.29245/2578-3009/2021/3.1221

**Published:** 2021-08-19

**Authors:** Lisa M. James, Apostolos P. Georgopoulos

**Affiliations:** 1 The HLA Research Group, Brain Sciences Center, Department of Veterans Affairs Health Care System, Minneapolis, MN, 55417, USA; 2 Department of Neuroscience, University of Minnesota Medical School, Minneapolis, MN 55455, USA; 3 Department of Psychiatry, University of Minnesota Medical School, Minneapolis, MN 55455, USA; 4 Department of Neurology, University of Minnesota Medical School, Minneapolis, MN 55455, USA

**Keywords:** Motor neuron diseases, Human leukocyte antigen, Epidemiology, Immunity, Genetics

## Abstract

Very few studies have evaluated associations of human leukocyte antigen (HLA) with motor neuron diseases (MND). Using an immunogenetic epidemiological approach, we identified a population-level HLA profile for MND by evaluating the correlations between the population frequencies of 127 HLA Class I and II alleles and the population prevalence of MND in 14 Continental Western European countries. The results demonstrated that significantly more HLA alleles, particularly for Class I, were negatively associated with the population prevalence of MND, suggesting a preponderance of protective vs susceptibility effects. The findings add to the limited literature implicating HLA in MND and considering the role of HLA in immune system responses to pathogens, suggest a potential influence of pathogens in MND.

## Introduction

Motor neuron diseases (MND) are a highly disabling group of neurodegenerative diseases characterized by upper and/or lower motor neuron degeneration. Amyotrophic lateral sclerosis (ALS), which is the most common MND and the most extensively studied, initially involves muscle weakness or stiffness that progresses to gradual loss of voluntary movement with fatality typically occurring within a few years of onset^1^. Neuropathological features include loss of motor neurons as well as cytoplasmic inclusions that mirror those seen in frontotemporal dementia^2^. Indeed, as ALS progresses, cognitive symptoms often emerge with varying degrees of impairment up to and including dementia, commonly of the frontotemporal type^3,4^. Notably, the course, phenotype, and survival time of ALS have been shown to vary geographically in relation to population ancestral origin, pointing towards a modulatory influence of genetic and environmental factors that vary by population^5^. A number of genes have been implicated in ALS, many of which overlap with frontotemporal dementia^6–8^. Still, a significant percent of genetic influence of ALS remains unknown, especially in the case of sporadic ALS^6^. Environmental contributors to ALS are similarly uncertain. Several risk factors including smoking, physical activity, environmental and occupational exposures, head injuries, and diet have been investigated with varying degrees of support^9,10^. There is increasing evidence implicating microorganisms (e.g., viruses, bacteria) in ALS pathogenesis^11^. With regard to other MND, all of which are relatively rare, there is considerable heterogeneity in terms of signs, symptoms, and prognosis^12^. Furthermore, with the exception of spinal muscular atrophy and hereditary spastic paraplegia which are known to have a genetic basis, the cause of other motor neuron diseases is largely unknown^12,13^.

In light of the largely undetermined genetic influence on ALS and other motor neuron diseases and the potential etiological involvement of microorganisms, we focused here on the immunogenetic influence of human leukocyte antigen (HLA), a region of genes on chromosome 6 that are involved in immune response to foreign antigens. The two main classes of HLA – Class I (HLA-A, B, -C) and Class II (HLA-DR, -DQ, DP) - play a critical role in elimination of foreign antigens. Class I presents intracellular antigen peptides to CD8+ cytotoxic T cells which signals destruction of infected cells. Class II presents endocytosed extracellular antigen peptides to CD4+ T cells to promote B-cell mediated antibody production and adaptive immunity. A limited number of studies, largely using low-resolution HLA typing, have evaluated the influence of HLA on ALS with inconsistent findings^14^. A recent review of the literature indicated primarily Class I associations with ALS^14^; specifically, HLA-A*03, A*02, A*28; B*40, B*35, and C*04 have been found to promote susceptibility whereas A*09 is protective. A recent study in a Chinese population reported risk associated with a single nucleotide polymorphism in the DR gene, suggesting a role for HLA Class II in ALS^15^. These findings suggest an immunogenetic component to ALS; however, further study of HLA associations with ALS and other MND is warranted. The highly polymorphic nature of HLA presents a challenge in terms of identifying specific alleles that may be associated with rare diseases such as MNDs at the individual level. Therefore, we are utilizing a population immunogenetic approach to identify an HLA profile with regard to MND prevalence to better understand risk and protection associated with a wide range of HLA alleles. We have used a similar approach to identify HLA profiles for dementia, Parkinson’s disease, multiple sclerosis, and Type 1 diabetes^16–20^. This approach takes advantage of the population heterogeneity of HLA and utilizes high-resolution HLA genotyping to determine HLA alleles that are presumed to be protective (i.e., negatively associated) or susceptible (i.e., positively correlated) with regard to the population prevalence of a disease.

## Materials and Methods

### Prevalence of MND

The population prevalence of MND was computed for each of the following 14 countries in Continental Western Europe: Austria, Belgium, Denmark, Finland, France, Germany, Greece, Italy, Netherlands, Portugal, Norway, Spain, Sweden, and Switzerland. Specifically, the total number of people with MND in each of the 14 Continental Western European countries as determined by the Global Burden of Disease study^21^ was divided by the total population of each country in 2016 (Population Reference Bureau)^22^ and expressed as a percentage. The Global Burden of Disease study included ALS, spinal muscular atrophy, hereditary spastic paraplegia, primary lateral sclerosis, progressive muscular atrophy, and pseudobulbar palsy in its evaluation of the population characteristics of MND. We have previously shown that life expectancy for these countries is virtually identical^17^; therefore, life expectancy was not included in the current analyses.

### HLA

The frequencies of all reported HLA alleles of classical genes of Class I (A, B, C) and Class II (DPB1, DQB1, DRB1) for each of the 14 Continental Western European countries were retrieved from the website allelefrequencies.net (Estimation of Global Allele Frequencies^23,24^) on October 20, 2020. There was a total of 2746 entries of alleles from the 14 Continental Western European countries, comprising 844 distinct alleles. Of those, 127 alleles occurred in 9 or more countries and were used in further analyses. This criterion is somewhat arbitrary but reasonable, since it encompasses ≥ 64.3% (≥ 9/14) of the whole sample of 14 countries. In addition, it was partially validated in a previous study^16^, where HLA-disease associations for dementia and Parkinson’s disease were congruent across a range of sample sizes.

The distribution of those alleles to the HLA classes and their genes is given in Table 1.

### Data analysis

HLA profiles for MND were derived as described previously for other diseases^16–20^. Briefly, the prevalence of MND in a country was computed as the fraction of total country population and was expressed as a percentage. MND prevalences were natural-log transformed and the Pearson correlation coefficient, r, between MND prevalence and the population frequency of each one of the 127 HLA alleles above calculated and Fisher z–transformed^25^ to normalize its distribution:

(1) $${\text{r}}^{\prime }\text{=atanh(r)}$$

The MND HLA profile consisted of 127 values of ${\text{r}}^{\prime }$. The effects of HLA Class and gene (within a class) on ${\text{r}}^{\prime }$ were evaluated using a univariate analysis of variance (ANOVA). Finally, differences in the proportions of the counts of negative and positive ${\text{r}}^{\prime }$ were evaluated using the Wald H0 statistic for comparing proportions of independent samples. Statistical analyses were performed using the IBM–SPSS package (IBM SPSS Statistics for Windows, Version 26.0, 64–bit edition. Armonk, NY: IBM Corp; 2019) and Intel FORTRAN (Microsoft Visual Studio Community Version 16.8.3; Intel FORTRAN Compiler 2021).

## Results

**Figure 1.**
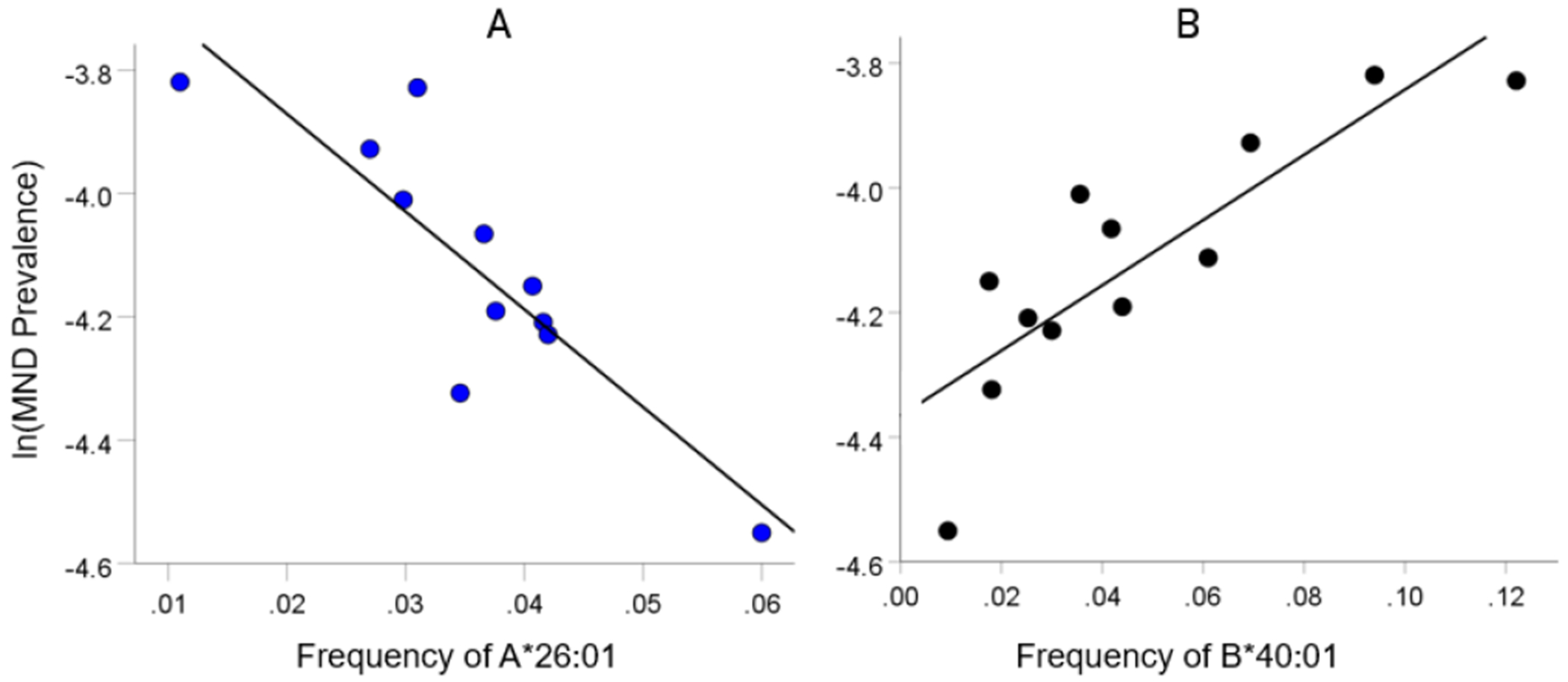
Example from a presumed protective HLA allele (A*26:01) and a presumed susceptibility allele (B*40:01) for MND. A, log-transformed MND prevalence (%) for 11 CWE countries is plotted against the corresponding frequency of the A*26:01 (P =0.0005). B, log-transformed MND prevalence (%) for 12 CWE countries is plotted against the corresponding frequency of the B*40:01 (P =0.0005).

**Figure 2.**
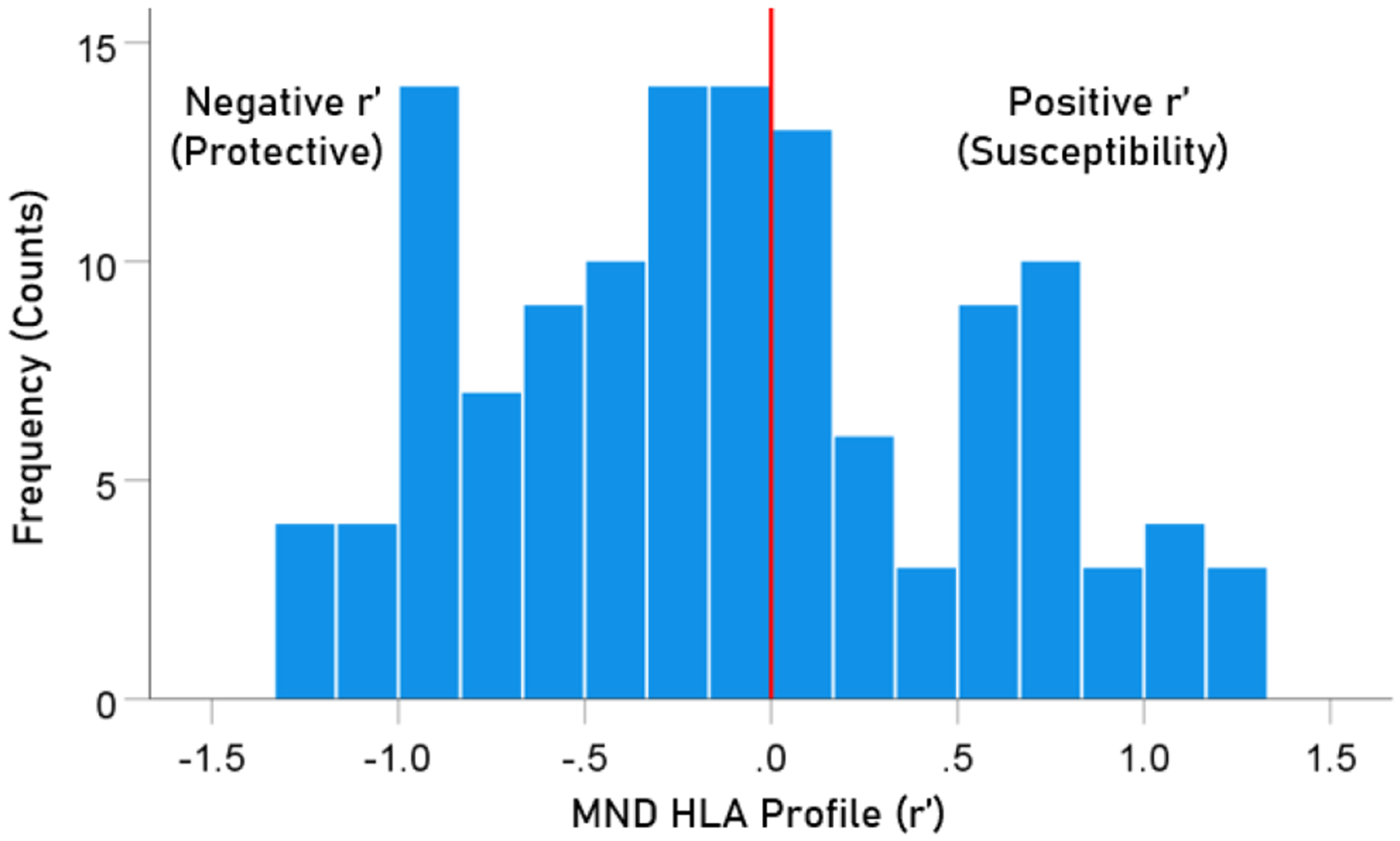
Frequency distribution of MND HLA profile (N = 127).

As mentioned above, the MND HLA profile consists of correlations between allele frequency and disease prevalence, suitably Fisher z-transformed (Equation 1) to normalize their distribution for further analyses. We showed previously^17^ that dementia prevalence varies in an exponential fashion with allele frequency, such that the logarithm of disease prevalence is a linear function of allele frequency. We found the same relation here between MND prevalence and HLA allele frequency. Two examples are illustrated in Figs. 1 and 2, namely for a presumed MND protective allele (A&26:01) and a susceptibility allele (B*40:01) (Fig. 1A and B, respectively).

## HLA-MND profile

The frequency distribution of alleles in the HLA MND profile (Table 2) is shown in Fig. 2. There were 76/127 (59.8%) negative (protective) alleles and 51/127 (40.2%) positive (susceptibility) alleles. These percentages differed significantly from the null hypothesis of 50% (P = 0.027, two-sided one-sample binomial test; z = 2.218).

**Figure 3.**
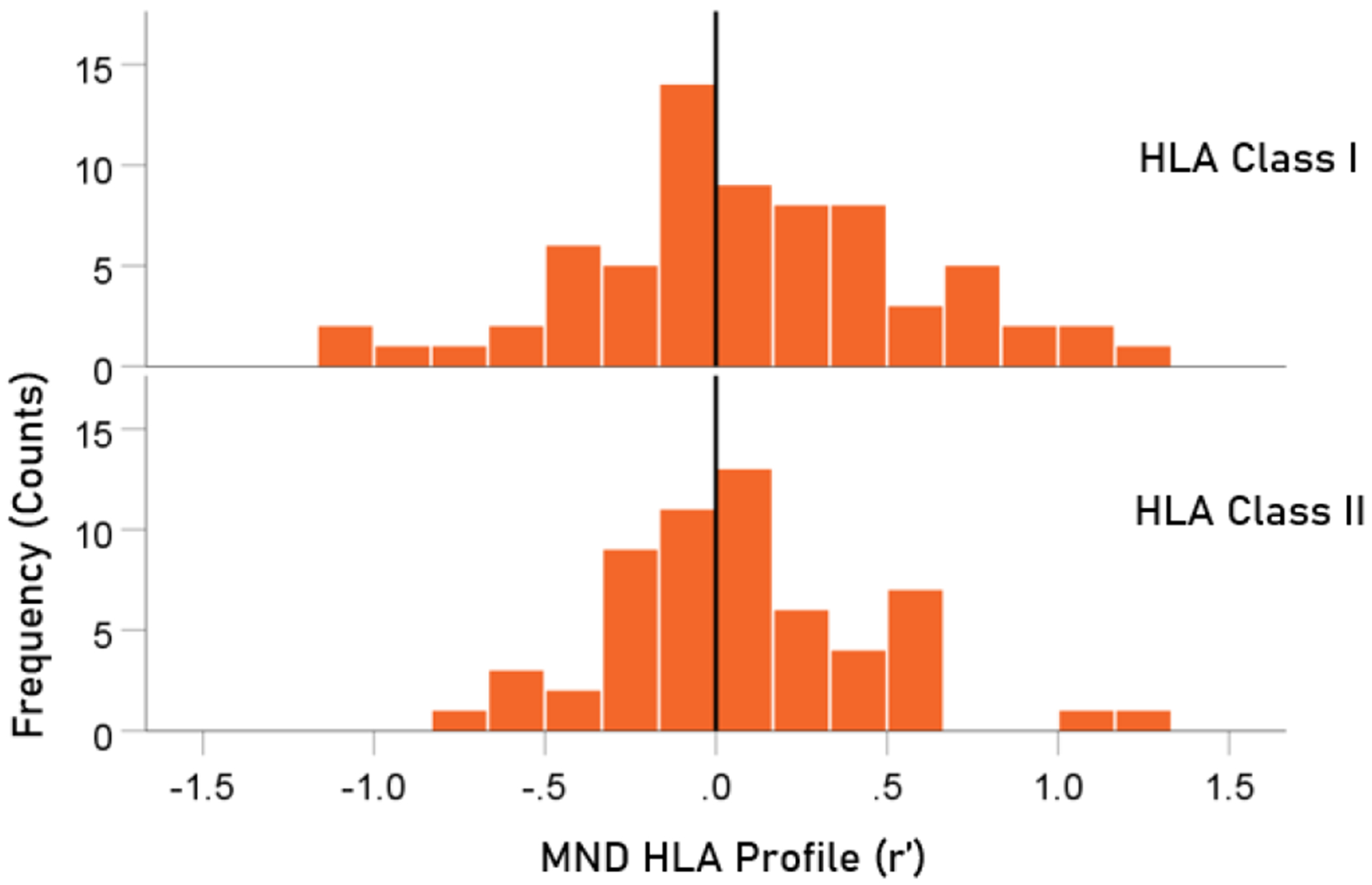
HLA Class distributions of MND HLA profile. N = 69 alleles for Class I and 58 alleles for Class II.

The distributions of ${\text{r}}^{\prime }$ for Class I and II are shown in Fig. 3. There were 69/127 (54.3%) ${\text{r}}^{\prime }$ in Class I and 58/127 (46.7%) in Class II; these percentages did not differ significantly from the 50-50% null hypothesis (P = 0.329, two-sided one-sample binomial test; z = 0.976). For Class I, there were 45/69 (65.2%) negative (protective) and 24/69 (34.8%) positive (susceptibility) values, respectively; these percentages differed significantly from the 50-50% null hypothesis (P = 0.011, two-sided one-sample binomial test; z = 2.528). For Class II, there were 31/58 (53.4%) negative and 27/58 (46.6%) positive values, respectively; these percentages did not differ significantly from the 50-50% null hypothesis (P = 0.599, two-sided one-sample binomial test; z = 0.535).

## Analysis of strength of ${\text{r}}^{\prime }$

There were no statistically significant differences in the strength of ${\text{r}}^{\prime }\left(\left|{\text{r}}^{\prime }\right|\right)$ between the protective and susceptibility groups for either HLA class or gene (within a class) (P>0.05 for all comparisons, independent samples t-test).

## Discussion

In the present study we used an immunogenetic epidemiological approach across 14 countries in Continental Western Europe to identify a population–level HLA profile consisting of protective and susceptibility alleles for MND. Few prior studies have evaluated HLA associations with ALS or other MND and most of those have focused on Class I alleles. Here we identified robust HLA-MND associations particularly involving Class I alleles but also several strong associations with Class II alleles. These findings, which suggest a broader influence of HLA on MND beyond the small number of Class I alleles that have been previously documented to be associated with MND, are discussed below.

Nearly 60% of the HLA alleles investigated here were negatively associated with the population prevalence of MND and presumed to be protective. Moreover, for Class I alleles in particular there were significantly more protective alleles than susceptibility alleles; Class II alleles did not significantly differ in terms of protection vs susceptibility. The relative rarity of MNDs^26^ may be partially related to the preponderance of protective (i.e., negatively correlated) alleles observed in the present study. These findings notably stand in contrast to prior research using the same approach that demonstrated a preponderance of susceptibility alleles in both dementia and Parkinson’s disease, two conditions that are much more frequent than MND^16^. Previous research has documented protective effects for A*09^27^. The current analyses included only those alleles that were present in at least 9 of the 14 countries; thus, A*09 was not included in the present analyses. Here, the strongest protective effects (i.e., negative correlations with the population prevalence of MND) were found for three Class I alleles (A*26:01, A*32:01, C*13:02) and for two Class II alleles (DRB1*15:02 and DQB1*05:02). In light of the evolutionary role of HLA in host protection from foreign antigens such as viruses and bacteria via T- cell and B-cell mediated immune mechanisms, we presume that the protective effects of Class I and Class II HLA alleles observed here are related to elimination of pathogens that have been implicated in MND^11^.

We have previously proposed that pathogen exposure in the absence of protective HLA results in persistent antigens that may promote disease through direct damage to cells and/or, in the presence of HLA susceptibility alleles, autoimmunity due to chronic inflammation^16^. With regard to ALS, accumulating evidence indicates autoimmune mechanisms against motor nerve terminals and voltage-dependent calcium channels result in apoptosis and neuronal death^28^. In addition, increased Class II HLA-DR expression in peripheral nerves of ALS patients has been suggested to reflect an autoimmune mechanism targeting Schwann cells^29^. Furthermore, evidence of inflammation in ALS as indicated by an increase in the number of microglial cells and reactive microglia displaying high levels of Class I and Class II HLA^30,31^. In the present study, both Class I and Class II HLA were associated with increased population prevalence of MND. The strongest positive associations were found for Class I alleles including B*07:02, B*15:01, B*37:01, B*40:01, C*03:03. Previous research has identified increased risk associated with B*40 as well as A*02, A*03, A*28, B*35, and C*04^12^, most of which were also associated with population susceptibility in the current study.

ALS, the most common MND, is considered to be part of a continuum with other neurodegenerative diseases including frontotemporal dementia and Parkinson’s disease^10^. The current findings suggest that immunogenetic mechanisms are part of that continuum. Indeed, similar to the present findings, HLA has been implicated in the population prevalence of several other neurodegenerative conditions including dementia, Parkinson’s disease, and multiple sclerosis^16–20^. Considering the evolutionary role of HLA in immune response to foreign antigens, these studies suggest a common role of foreign antigens (e.g., viruses, bacteria) in these neurodegenerative conditions. Work is ongoing in our lab to evaluate in silico the binding affinity of candidate antigens with specific HLA alleles^32,33^.

Identification of HLA-MND associations at the individual level is hampered by the infrequency of MND and the extreme polymorphism of HLA; prohibitively large samples of MND patients would be required to evaluate MND associations with the wide range of HLA alleles investigated here. In addition, the few prior studies evaluating HLA in relation to MND have often been limited by reliance on low-resolution HLA typing which masks important protein-level differences in disease associations. For instance, in the present analyses, DRB1*15:01 was positively associated with MND whereas DRB1*15:02 was negatively associated with MND. Protein level differences have been shown to alter the binding groove, shaping the repertoire of antigens that can bind and stimulate an immune response^34^. The current population level approach permits evaluation of numerous high–resolution Class I and Class II HLA alleles with MND prevalence. In addition, inclusion of data from several countries increases allele diversity and regional generalizability of the findings. That being said, the HLA-MND associations observed in these 14 Continental Western European countries may not extend to other regions given population variability in HLA^35,36^. In addition, our analyses are based on the Global Burden of Disease Study population counts of several conditions classified together as MND; however, HLA associations with each specific MND may vary and disease-specific HLA associations are not evaluated here. Finally, the analyses are based on correlations between the population frequency of HLA alleles and the population prevalence of MND; while the results provide compelling evidence of robust HLA-MND associations at the population level, additional studies are warranted to determine causal associations. We assume that HLA-MND associations implicate pathogens as a contributor to MND given the evolutionary role of HLA in pathogen elimination; however, the influence of specific pathogens on HLA-MND associations remains to be determined.

## Conclusion

Compared to other neurodegenerative conditions research evaluating HLA associations with MND is limited. Here we evaluated immunogenetic influences on MND at the population level. The findings support a role of Class I and Class II HLA in the population prevalence of MND and extend the existing literature to identify a number of susceptibility and protective alleles. Considering the role of HLA in immune system responses to foreign antigens, these findings point to a potential contributory role of pathogens in MND.

## Figures and Tables

**Table 1:** Distribution of 127 HLA alleles analyzed to Class and Genes.

	Class I (N = 69)			Class II (N = 58)		
Gene	A	B	C	DPB1	DQB1	DRB1
Count	20	36	13	15	14	29

**Table 2.** HLA profile of MND. The signed z-transformed correlation coefficient (${\text{r}}^{\prime }$) between 127 HLA alleles and ln (MND) prevalence. N denotes the number of CWE countries from which ${\text{r}}^{\prime }$ was calculated.

	Allele	Class	N	${\text{r}}^{\prime }$(MND)
1	A*01:01	I	11	−.360
2	A*02:01	I	11	0.402
3	A*02:05	I	9	−0.193
4	A*03:01	I	11	1.080
5	A*11:01	I	11	−0.682
6	A*23:01	I	11	−0.636
7	A*24:02	I	11	0.158
8	A*25:01	I	12	0.083
**9**	A*26:01	**I**	**11**	**−1.322**
10	A*29:01	I	11	−0.016
11	A*29:02	I	11	0.003
12	A*30:01	I	11	−0.426
13	A*30:02	I	12	−0.180
14	A*31:01	I	9	0.929
**15**	A*32:01	**I**	**12**	**−1.282**
16	A*33:01	I	10	−0.116
17	A*33:03	I	9	−0.957
18	A*36:01	I	10	−0.354
19	A*68:01	I	11	−0.094
20	A*68:02	I	10	−0.132
**21**	B*07:02	**I**	**10**	**1.118**
22	B*08:01	I	12	0.407
23	B*13:02	I	11	−0.269
24	B*14:01	I	11	−0.040
25	B*14:02	I	10	−0.104
**26**	B*15:01	**I**	**10**	**1.187**
27	B*15:17	I	9	−0.159
28	B*15:18	I	9	−0.272
29	B*18:01	I	12	−0.870
30	B*27:02	I	10	0.182
31	B*27:05	I	12	0.672
32	B*35:01	I	11	0.025
33	B*35:02	I	9	−0.577
34	B*35:03	I	9	−1.046
35	B*35:08	I	9	−0.830
**36**	B*37:01	**I**	**10**	**1.180**
37	B*38:01	I	9	−0.937
38	B*39:01	I	11	−0.356
39	B*39:06	I	9	−0.147
**40**	B*40:01	**I**	**12**	**1.247**
41	B*40:02	I	12	0.294
42	B*41:01	I	11	−0.321
43	B*41:02	I	10	−0.585
44	B*44:02	I	12	0.064
45	B*44:03	I	12	−0.154
46	B*44:05	I	9	−0.843
47	B*45:01	I	10	0.219
48	B*47:01	I	11	−0.193
49	B*49:01	I	11	−0.726
50	B*50:01	I	10	−0.331
51	B*51:01	I	10	−0.860
52	B*52:01	I	10	−0.871
53	B*55:01	I	11	0.324
54	B*56:01	I	9	0.660
55	B*57:01	I	12	−0.594
56	B*58:01	I	9	−0.652
57	C*01:02	I	9	0.510
**58**	C*03:03	**I**	**9**	**1.123**
59	C*04:01	I	9	−0.762
60	C*05:01	I	9	0.509
61	C*06:02	I	9	−0.542
62	C*07:01	I	9	0.027
63	C*07:02	I	9	1.098
64	C*07:04	I	9	−0.589
65	C*12:02	I	9	−0.897
**66**	C*12:03	**I**	**9**	**−1.158**
67	C*14:02	I	9	−0.943
68	C*15:02	I	9	−0.909
69	C*16:01	I	9	−0.0005
70	DPB1*01:01	II	11	0.946
71	DPB1*02:01	II	11	−0.934
72	DPB1*02:02	II	10	−0.148
73	DPB1*03:01	II	11	0.165
74	DPB1*04:01	II	11	0.545
75	DPB1*04:02	II	11	−0.190
76	DPB1*05:01	II	11	0.747
77	DPB1*06:01	II	10	0.289
78	DPB1*09:01	II	9	−0.183
79	DPB1*10:01	II	10	−0.567
80	DPB1*11:01	II	9	0.133
81	DPB1*13:01	II	10	−0.825
82	DPB1*14:01	II	11	−0.918
83	DPB1*17:01	II	9	−0.315
84	DPB1*19:01	II	11	0.415
85	DQB1*02:01	II	12	0.533
86	DQB1*02:02	II	11	−0.278
87	DQB1*03:01	II	13	−1.059
88	DQB1*03:02	II	13	0.981
89	DQB1*03:03	II	13	0.722
90	DQB1*04:02	II	13	0.800
91	DQB1*05:01	II	13	0.154
**92**	DQB1*05:02	**II**	**10**	**−1.206**
93	DQB1*05:03	II	12	−0.612
94	DQB1*06:01	II	11	−0.371
95	DQB1*06:02	II	14	0.733
96	DQB1*06:03	II	13	0.310
97	DQB1*06:04	II	12	0.048
98	DQB1*06:09	II	9	−0.022
99	DRB1*01:01	II	14	0.538
100	DRB1*01:02	II	11	−0.477
101	DRB1*01:03	II	11	−0.389
102	DRB1*03:01	II	13	−0.004
103	DRB1*04:01	II	13	0.666
104	DRB1*04:02	II	11	−0.732
105	DRB1*04:03	II	12	−0.952
106	DRB1*04:04	II	13	0.833
107	DRB1*04:05	II	9	−0.189
108	DRB1*04:07	II	12	−0.145
109	DRB1*04:08	II	9	0.798
110	DRB1*07:01	II	12	−0.396
111	DRB1*08:01	II	13	0.779
112	DRB1*08:03	II	11	0.107
113	DRB1*09:01	II	12	0.566
114	DRB1*10:01	II	14	0.004
115	DRB1*11:01	II	14	−0.354
116	DRB1*11:02	II	12	−0.252
117	DRB1*11:03	II	12	−1.002
118	DRB1*11:04	II	12	−0.781
119	DRB1*12:01	II	13	0.614
120	DRB1*13:01	II	14	0.694
121	DRB1*13:02	II	14	0.160
122	DRB1*13:03	II	10	−0.862
123	DRB1*13:05	II	10	−0.214
124	DRB1*14:01	II	14	−0.471
125	DRB1*15:01	II	13	0.689
**126**	DRB1*15:02	**II**	**10**	**−1.206**
127	DRB1*16:01	II	10	−0.899

Note. Strongest associations are denoted in bold.
